# A computational model of the epidermis with the deformable dermis and its application to skin diseases

**DOI:** 10.1038/s41598-021-92540-1

**Published:** 2021-06-24

**Authors:** Kota Ohno, Yasuaki Kobayashi, Masaaki Uesaka, Takeshi Gotoda, Mitsuhiro Denda, Hideyuki Kosumi, Mika Watanabe, Ken Natsuga, Masaharu Nagayama

**Affiliations:** 1 Department of Data Science for Business Innovation, Faculty of Science and Engineering, Chuo University, Tokyo, Japan; 2 Research Center of Mathematics for Social Creativity, Research Institute for Electronic Science, Hokkaido University, Sapporo, Japan; 3 Graduate School of Mathematical Sciences, The University of Tokyo, Tokyo, Japan; 4 Graduate School of Mathematics, Nagoya University, Nagoya, Japan; 5 Shiseido Global Innovation Center, Yokohama, Japan; 6 Department of Dermatology, Hokkaido University Faculty of Medicine and Graduate School of Medicine, Sapporo, Japan

**Keywords:** Computational models, Applied mathematics, Skin diseases

## Abstract

The skin barrier is provided by the organized multi-layer structure of epidermal cells, which is dynamically maintained by a continuous supply of cells from the basal layer. The epidermal homeostasis can be disrupted by various skin diseases, which often cause morphological changes not only in the epidermis but in the dermis. We present a three-dimensional agent-based computational model of the epidermis that takes into account the deformability of the dermis. Our model can produce a stable epidermal structure with well-organized layers. We show that its stability depends on the cell supply rate from the basal layer. Modeling the morphological change of the dermis also enables us to investigate how the stiffness of the dermis affects the structure and barrier functions of the epidermis. Besides, we show that our model can simulate the formation of a corn (clavus) by assuming hyperproliferation and rapid differentiation. We also provide experimental data for human corn, which supports the model assumptions and the simulation result.

## Introduction

Skin is a pivotal organ that prevents water loss and protects us from various external pathogens and stimuli^1,2^. Stem cells in the basal layer continuously supply cells into suprabasal layers, consisting of the spinous, granular, and cornified layers from below. The cornified layer, the outermost part of the skin, consists of flat, regularly stacked cornified cells and the lipids filling the spaces between them, and its organized structure is responsible for epidermal barrier functions. Therefore, to understand epidermal homeostasis and its barrier function, one has to elucidate how the organized layer structure is maintained.

Mathematical modeling is a valuable tool to investigate the emergence of epidermal homeostasis as a complex phenomenon. Among others, agent-based models have been widely adopted to study homeostatic properties of the epidermis^3–6^. One advantage of using agent-based models is that they can easily incorporate various features, such as stem cell dynamics, differentiation, lipid production and secretion, and cell morphology. Pathological states of the skin can also be easily created, enabling one to study wound healing^7–9^ and the development of psoriasis^10^. An integrated model of the epidermis that includes all features relevant to epidermal homeostasis would be desired to simulate various skin diseases and understand their mechanisms.

One factor that needs to be taken into account to have such an integrated model is a localized layer of calcium ions beneath the cornified layer, which could affect barrier functions^11–18^. Mathematical models were proposed for the calcium gradient in the epidermis^19,20^, as well as localized calcium excitation in cultured keratinocytes^21^, and the effect of the calcium layer on the epidermal structure was investigated^22,23^. The effect of calcium was further studied by using agent-based models^24,25^. By introducing an agent-based model of the epidermis, we suggested that the acceleration of differentiation due to calcium ions could stabilize the boundary between the granular and the cornified layers^26^.

Another important factor is the shape of the dermis, which could affect the spatial patterns of cell supply from the basal layer. The effect of dermal shape on the thickness of the epidermis was studied using a rigid, undulating dermis^27^, which suggested that an increase of the surface area due to dermal undulations could lead to an increase of epidermal thickness; This effect has been supported by an experiment^28^. Dynamical processes of dermal deformation were also studied: We proposed an agent-based model with the deformable dermis, which successfully simulated upward protrusions of the dermis starting from a flat dermis^29^, as observed in real human epidermis. It is well known that growing tissues can develop a spatial structure due to the buckling instability^30–38^; our model has revealed that the structure could also affect the spatial patterning of stem cells.

Our two previous models mentioned above are complementary: the epidermal model^26^ has not taken into account deformability of the dermis; The dermal deformation model^29^ has taken into account only the dermis, the basement membrane, and the basal layer, disregarding the suprabasal layers. In this work, we integrate these two models into a unified model that can simulate epidermal homeostasis with the deformable dermis. This model includes important aspects for simulating the maintenance of the epidermis, such as cell division in the basal layer, calcium-dependent cell differentiation, flattening of cell shape during differentiation, secretion of lipids, desquamation, and the development of a spatial structure of the dermis due to cell division. By numerical simulations, we demonstrate that the model can produce a stable epidermis with well-organized layer structures. Extensive numerical investigations reveal that the stability of the layer structure depends on the supply rate of cells from the basal layer. Besides, by controlling the stiffness of the dermis, we show how the hardening of the dermis affects epidermal homeostasis. Finally, we show that our model can be used to simulate a skin disease that causes morphological changes in both the epidermis and the dermis, such as the corn (clavus). We also show an experimental result of the corn formation and compare it with the simulation result.

## Results

### Overview of the computational model

We consider the system composed of the dermis, the basement membrane, and the epidermis. The dermis is a soft elastic substrate, which is modeled by particles adhesive to each other. The stiffness of the dermis is controlled by modifying the adhesion strength. The basement membrane is modeled by particles connected in the form of a triangular lattice. The lattice edges are assigned stretching and bending energies so that the membrane exhibits elasticity. Membrane particles are adhesive to dermal particles. Epidermal cells are represented by spheroids, whose flattening rate depends on differentiation.

The basal layer is defined as a monolayer of basal cells, which are stem cells or transit-amplifying (TA) cell. Stem cells are strongly bound to the basement membrane, whereas TA cells are weakly bound. Cells passively move due to the pressure created by repeated cell division, which causes TA cells to leave the basement membrane. Stem cells divide an infinite number of times, whereas TA cells divide a finite number of times, $N_{\mathrm {div}}$. Both cells follow a stochastic cell cycle, with the deterministic period $T_{\mathrm {div}}$. TA cells not bound to the basement membrane are regarded as differentiated, which constitutes the suprabasal layer. Continuous cell division in the basal layer causes the migration of cells towards the upper layers.

A cell is assigned a state variable; it starts to increase when differentiated, with the increase accelerated by calcium ions and stimulants released by the cornified cells. Cell type changes as spinous, granular, or cornified in this order as the state variable increases. Lipids are produced inside granular cells and released when calcium ions increase, which typically occurs at cornification. After cornification, the cell undergoes desquamation, i.e., peels off from the bulk (computationally, it is removed from the system) controlled by corneodesmosomes, cell-junction structures specific to cornified cells.

**Figure 1:**
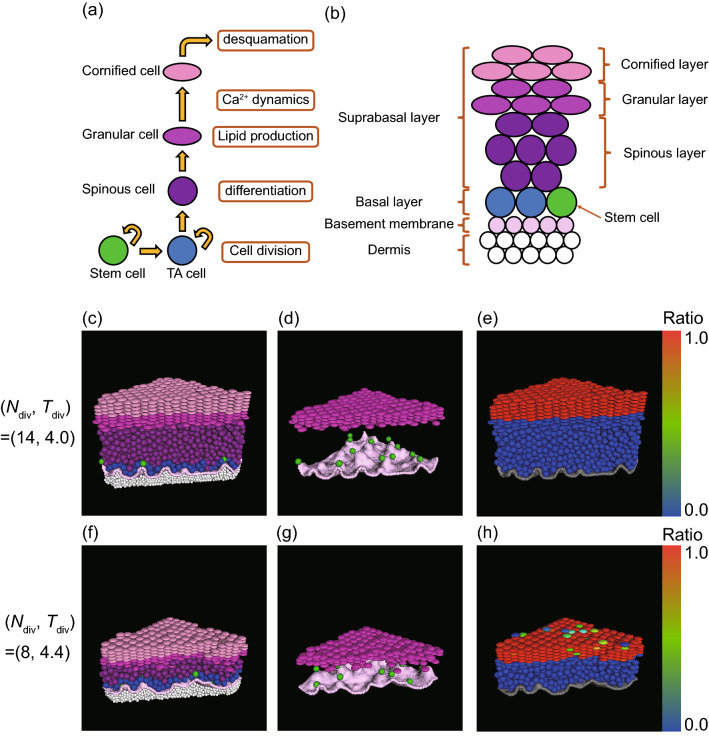
Simulation for the development of the epidermis. (**a**) Modeling of cell dynamics. (**b**) Schematic representation of the dermis, the basement membrane, and different layers of the epidermis. (**c**)–(**h**) Simulation snapshots for two different sets of $N_{\mathrm {div}}$ (maximum number of cell divisions) and $T_{\mathrm {div}}$ (cell division period): (**c**–**e**) $(N_{\mathrm {div}}, T_{\mathrm {div}}) = (14, 4.0)$, (**f**–**h**) $(N_{\mathrm {div}}, T_{\mathrm {div}}) = (8.0, 4.4)$. (**c**), (**f**) Overview of the epidermal model, colored in the same way as (**b**): the dermis (white), the basement membrane (light pink), stem cells (green), TA cells (blue), spinous cells (purple), granular cells (dark magenta), cornified cells (dark pink). (**d**) and (**g**) are the same as (**c**) and (**f**), respectively, with only the basement membrane, stem cells, and granular cells are visualized. (**e**) and (**h**) are the same as (**c**) and (**f**), respectively, with cells colored according to the ratio of the lipid content to the maximum lipid production.

Hence, the model consists of equations of motion for dermal particles, membrane particles, and epidermal cells; cell division cycle; cell differentiation process; cell flattening during differentiation; lipid production; desquamation; and dynamics of calcium ions and stimulants. A schematic illustration is shown in Fig.  1(a). A full description of the mathematical model is given in Supplementary Information.

The scales of length and time in this model were determined so that the cell diameter is 10 $\upmu \hbox {m}$ and the time-span between cornification and desquamation is 14 days, whereby the turnover time for the whole epidermis is approximately 28 days.

### Maintenance of epidermal homeostasis

In the real epidermis, the barrier function is often evaluated by transepidermal water loss, the loss of body water through the epidermis. It is known that the water loss is prevented by regularly stacked cornified cells and intercellular lipids filling the spaces between them. Therefore, the barrier function requires a well-organized layer structure and sufficient lipid content. Hence we first checked whether the present model could produce an epidermis serving as a barrier both in terms of the structure and the lipid production.

We performed simulations with two different conditions by changing the maximum number of cell divisions $N_{\mathrm {div}}$ and the cell division period $T_{\mathrm {div}}$, which affects the cell supply rate from the basal layer. The results are shown in Fig. 1. Cell types and cell layers are depicted in Fig. 1(b). When a sufficient number of cells are continuously supplied from the basal layer, a fully developed epidermis was formed with clearly separated layers, consisting of spinous, granular, and cornified cells [Fig. 1(c)], The thickness of each layer was fairly uniform in space and stable in time [Fig. 1(c), (d); see also Fig. 4]. Columnar structures of vertically stacked cells were also observed in the granular layer and the cornified layer [Fig. 1(c), (d)]. The epidermal development was accompanied by the creation of upward protrusions in the initially flat dermis, with stem cells located at the tip of the protrusions [Fig. 1(d)]. We compared this result with another simulation in which the cell supply rate was reduced by choosing a smaller $N_{\mathrm {div}}$ value and a larger $T_{\mathrm {div}}$ value. In this case, we observed not only a decrease of thickness, as expected, but also the destabilization of the layer structure [Fig. 1(f)]. In particular, the boundary between the granular layer and the spinous layer became blurred due to isolated granular cells away from the bulk layer [Fig. 1(g)]. The effect of reduced cell supply was also found in the lipid production: When the cell supply is sufficient, intercellular lipids were sufficiently released from cornified cells [Fig. 1(e)]. When the cell supply was reduced, however, we observed insufficient lipid productions in a fraction of cells [Fig. 1(h)]. These results suggest that our model can produce a stable epidermal structure when cell supply from the basal layer is sufficient and that the reduced cell supply could affect both structural stability and the internal cell dynamics like lipid production.

### Evaluation of epidermal conditions by changing cell supply rate

**Figure 2:**
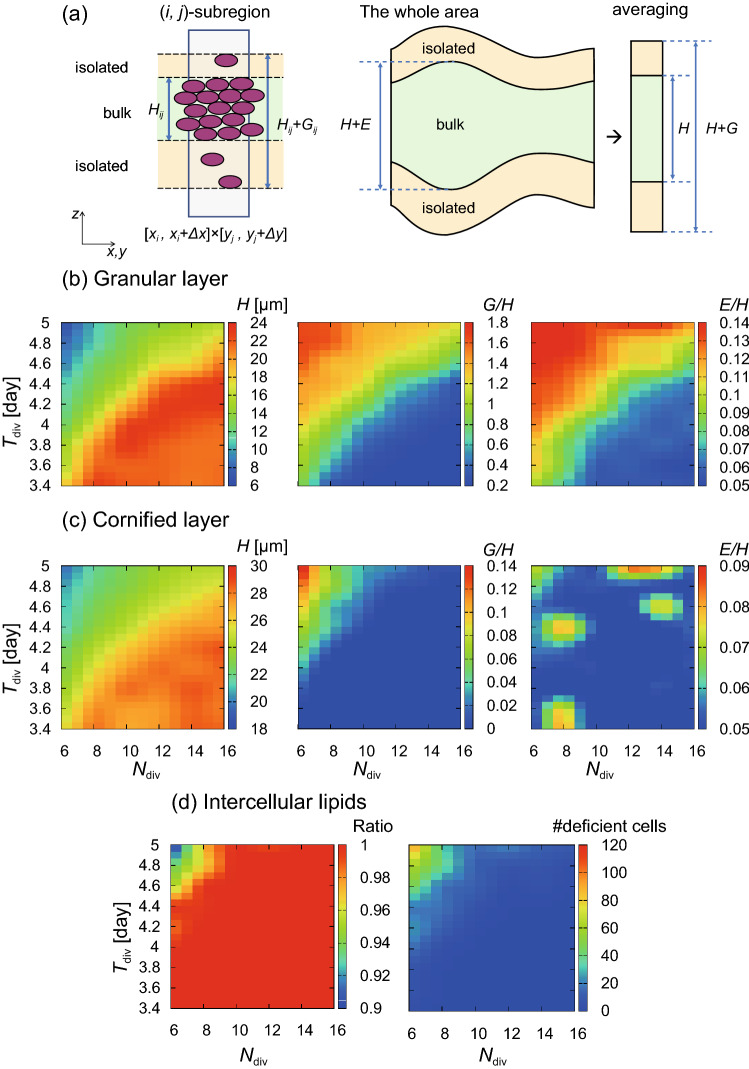
Evaluation of the epidermal structures and the lipid production as functions of $N_{\mathrm {div}}$ (maximum number of cell divisions) and $T_{\mathrm {div}}$ (cell division period). (**a**) Schematic illustration of the definition of the mean thickness *H*, the dispersion *G*, and the spatial variation *E*. (**b**) Thickness *H*, relative dispersion *G*/*H* normalized by thickness, and relative spatial variation *E*/*H* normalized by thickness for the granular layer. (**c**) *H*, *G*/*H*, and *E*/*H* for the cornified layer. (**d**) Ratio of the lipid content released from cornified cells to the maximum lipid production (left) and the number of lipid-deficient cornified cells with inadequate lipid production (less than 50% of the maximum) (right). All values in (**b**)–(**d**) are time-averaged over 280 days (approximately 10 turnovers). See Methods for precise definitions of the evaluation functions.

Then we investigated the effect of the cell supply rate on the epidermal structure and the lipid production more systematically by varying the parameters $N_{\mathrm {div}}$ and $T_{\mathrm {div}}$. We focus on the granular and the cornified layers. To evaluate the structure of these layers, we introduce the following measures: For each cell layer (granular or cornified), we define the thickness *H*; the dispersion *G*, and the spatial variation of the thickness *E*. A schematic illustration of these measures is given in Fig. 2(a) (Precise definitions are given in the Method): *H* is the thickness of the bulk; *G* expressed the largest vertical deviation of isolated cells from the bulk; and *E* represents the magnitude of modulations of the bulk thickness. These measures can capture different features: A cell layer with uniform thickness and a well-defined boundary with the adjacent cell layer should have small *G* and *E*.

Figure 2 shows the evaluation measures as a function of $N_{\mathrm {div}}$ vs $T_{\mathrm {div}}$. In the granular layer, the parameter space can be divided into two regions [Fig. 2(b)]: In the lower-right region, where the cell supply rate is large (with large $N_{\mathrm {div}}$ and small $T_{\mathrm {div}}$), the thickness *H* is high and both the dispersion *G* and the spatial variation *E* (normalized by the thickness *H*) are small, indicating a spatially uniform, well-defined granular layer. On the other hand, in the upper-left region, where the cell supply rate is small (with small $N_{\mathrm {div}}$ and large $T_{\mathrm {div}}$), the opposite tendency is observed, with small *H*, large *G*/*H*, and large *E*/*H*, indicating a thin bulk layer with large spatial variation of the thickness and with many isolated cells from the bulk. The same tendency is seen in the cornified layer [Fig. 2(c)]: in the lower-right region, the cornified layer also has large *H* and small *G* and *E*. We note, however, that the parameter region that produces large *G* and *E* values is narrower in the cornified layer than in the granular layer and that the magnitude of *G* and *E* is the larger in the granular layer, which suggests that the maintenance of the granular layer is more crucial for epidermal homeostasis. The same tendency is also found in the intercellular lipids. As shown in Fig. 2(d), the upper-left region of the $N_{\mathrm {div}}$-$T_{\mathrm {div}}$ space shows both the decrease of the mean lipids released from individual cells (normalized by maximum lipid production) and the increase of the number of lipid-deficient cornified cells, defined as cells with lipid production less than 50% of the maximum. The appearance of lipid-deficient cells is also visible in Fig. 1(h). These results suggest that sufficient cell supply is required for lipid production, as well as structural stability.

**Figure 3:**
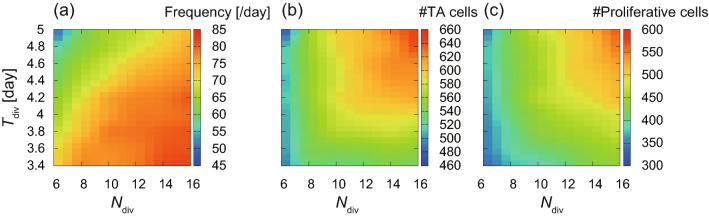
The ability of cell supply in the basal layer as a function of $N_{\mathrm {div}}$ and $T_{\mathrm {div}}$. (**a**) The frequency of cell division events in the basal layer per day. (**b**) The number of basal cells. (**c**) The number of proliferative cells.

We directly confirmed the relation between the cell supply from the basal layer and the parameters $N_{\mathrm {div}}$ and $T_{\mathrm {div}}$. Figure 3(a) shows the mean frequency of cell division events in the basal layer per day, which is low in the upper-left region, as expected. Note that the frequency is approximated by the number of proliferative cells divided by $T_{\mathrm {div}}$. The number of proliferative cells depends on the surface area of the basal layer, which also varies by these parameters. Figures 3(b) and 3(c) show the number of basal cells, which is proportional to the surface area of the basal layer, and the number of proliferative cells, respectively, which indicates that both the surface area and the number of proliferative cells increase as $N_{\mathrm {div}}$ increases. Note that the increase of $T_{\mathrm {div}}$, implying the reduced division frequency, does not necessarily reduce the surface area. On the contrary, the surface area increases as $T_{\mathrm {div}}$ increases for large $N_{\mathrm {div}}$ values, presumably because TA cells are more easily crowded out from the basal layer when cell division occurs more frequently.

### Effect of the stiffness of the dermis

**Figure 4:**
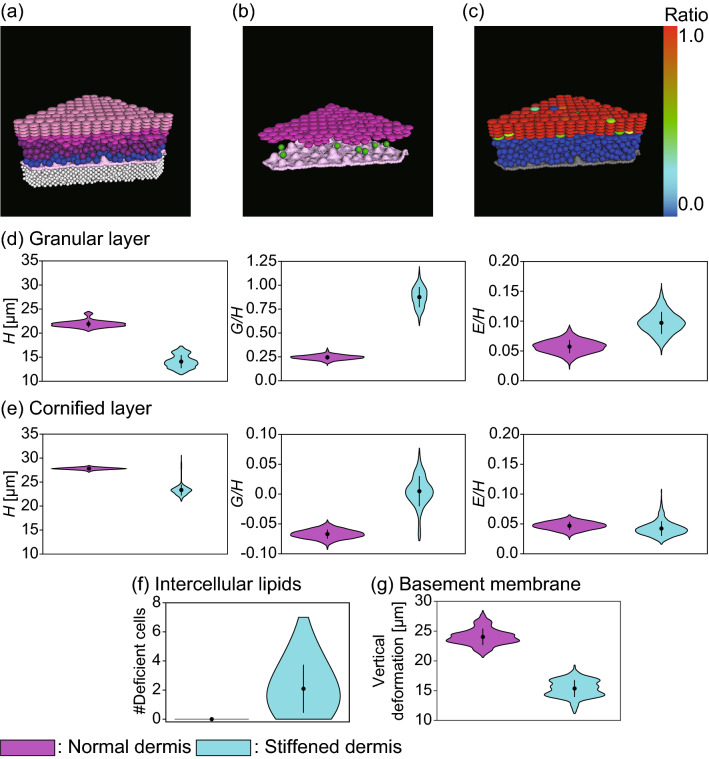
The effect of dermal stiffness. (**a**–**c**) Simulation with the stiffened dermis, presented in the same way as in Figs. 1(**a**–**c**) and (**d**–**g**). Comparison of the evaluation functions between the normal dermis and the stiffened dermis. (**d**) Evaluations of thickness *H*, normalized dispersion *G*/*H*, and normalized spatial variation *E*/*H* for the granular layer. (**e**) *H*, *G*/*H*, and *E*/*H* for the cornified layer. Note that the vartical scales for *G*/*H* are different between (**d**) and (**e**). (**f**) Number of cells with inadequate lipid production (less than 50% of the maximum). (**g**) Amplitude of the basement membrane deformation, defined by the difference between the maximum and minimum vertical displacements. Each violin plot contains 4001 data points within simulation time span $T=280$ days (approximately 10 turnovers). Mean values and standard deviations are shown by black dots and black vertical lines, respectively.

Next, to see how the structure and the barrier function would be affected by the deformability of the dermis, we performed a simulation by increasing the stiffness of the dermis and compared the result with the previous simulation in Fig. 1(c–e) as a normal condition. By stiffening the dermis, we observed that the layer structure was worsened and that lipid production was impaired [Fig. 4(a–c)]. Differences were especially notable in the granular layer [Fig. 4(d)]: the thickness *H* was greatly reduced, More isolated cells were observed (large *G*/*H*), and spatial variations were more enhanced (larger *E*/*H*). The differences become small but still recognizable in the cornified layer [Fig. 4(e)]. Temporal fluctuations of these quantities were also enhanced, as indicated by error bars in Fig. 4(d) and (e). Deficient cornified cells with inadequate secretion of lipids were also found [Fig. 4(f)].

The stiffening of the dermis directly affected the dermal shape, as diminished dermal undulations observed in Fig. 4(b). The vertical deformation of the basement membrane, defined by the difference between the maximum and the minimum vertical displacements, became small for the stiffened dermis [Fig. 4(g)]. Since the diminished undulations reduce the surface area, fewer basal cells are accommodated by the basement membrane, which results in the reduction of the cell supply rate. These results suggest that the stiffening of the dermis disrupts the epidermal structure and barrier functions because of the reduction of the cell supply rate.

### Simulation of the formation of a corn

**Figure 5:**
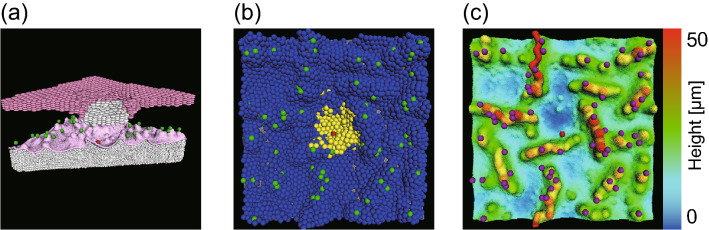
Simulation for the formation of a corn. (**a**) Cross-section. One stem cell (red) has an abnormality among normal stem cells (green). Cornified cells are colored white when produced from the abnormal stem cell and pink when produced from normal stem cells. (**b**) Top view of the basal layer. Yellow and blue cells are transit-amplifying (TA) cells that are originated from the abnormal stem cells and normal stem cells, respectively. (**c**) Top view of the basement membrane. The color indicates the vertical displacement of the baement membrane, measured from the lowest point. Stem cells are colored violet.

We wondered if our mathematical modeling could simulate human diseases by adjusting parameters. A corn (also termed clavus) is a well-demarcated and painful callus and typically develops on the plantar skin where the repeated friction or pressure is applied. We hypothesized that modulation of one stem cell is sufficient for corn development. We performed a simulation with the following modifications: We selected one stem cell as an abnormal cell; Those cells produced from this abnormal stem cell would divide twice as fast in the basal layer and differentiate twice as fast in the suprabasal layer as those produced from a normal stem cell. The system size was made larger than the previous simulations. As shown in Fig. 5(a), we found an inward intrusion of the cornified layer above the abnormal stem cell, where the intruding part of the cornified layer was mainly composed of cells produced by the abnormal stem cell [Fig. 5(a), colored red]. In the basal layer, fast-dividing cells formed a cluster around the abnormal stem cell [Fig. 5(b)], making a well-defined lesional area. Upward protrusions of the dermis were not observed in this lesional area, as in the non-lesional area, and the dermis in the lesional area was pushed downward, compared with the non-lesional area [Fig. 5(c)].

### Human corn morphology

**Figure 6:**
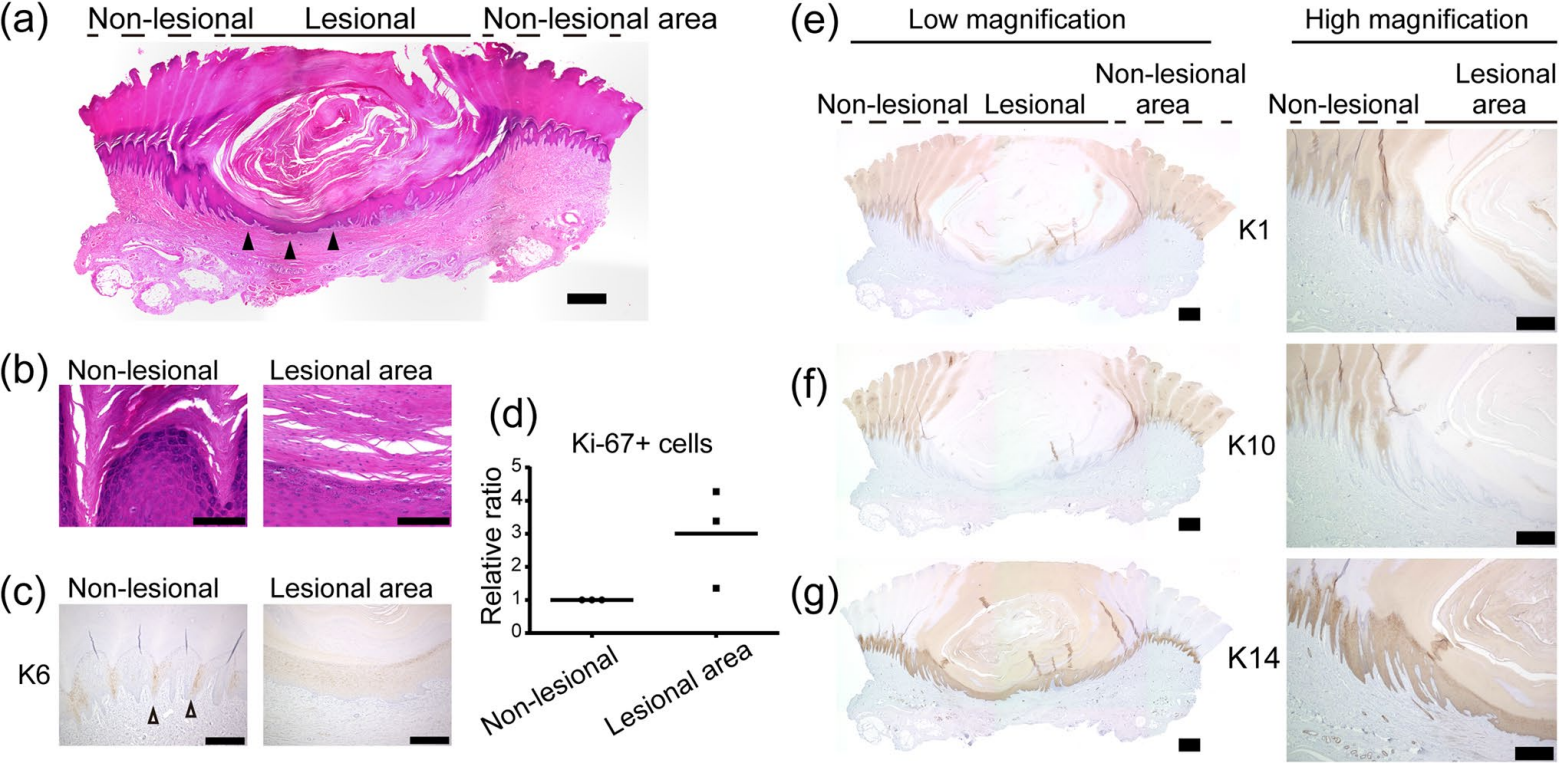
Histopathology of human corn (**a**, **b**) H&E staining. Scale bar: 1 mm (**a**) and 100 $\upmu \hbox {m}$ (**b**). (**c**) K6 labeling. Scale bar: 300 $\upmu \hbox {m}$. (**d**) Quantification of Ki-67+ cells in the epidermis. (**e**–**g**) K1, K10, and K14 labeling. Scale bar: 1 mm (low magnification) and 500 $\upmu \hbox {m}$ (high magnification).

Then we asked if the human corn reproduces the dynamics of proliferation and differentiation seen in the mathematical model (Fig. 5). We analyzed three corn specimens that were developed on the patients’ soles, and all of the samples shared similar findings. Hematoxylin and eosin (H&E) staining showed a massive hyperkeratosis (thickening of the cornified layer) in the lesional area of the corn [Fig. 6(a)], and the underlying epidermis was thinner than that of the non-lesional area (arrowheads). Parakeratosis (retention of the nuclei in the cornified layer) was also noted in the corn lesional area [Fig. 6(b)], suggesting the premature differentiation in the corn epidermis. Keratin 6 (K6) has been known to show an alternate expression in the palmoplantar epidermis^39^ [Fig. 6(c), non-lesional area, white arrowheads], but this pattern was absent in the corn [Fig. 6(c), lesional area]. Ki-67+ proliferative cells were more abundant in the corn than in the surrounding normal epidermis [Fig. 6(d)]. Besides, the epidermal differentiation markers (keratin 1 (K1) and 10 (K10) were absent, while the basal cell marker keratin 14 (K14) was retained even in the cornified layer in the corn [Fig. 6(e–g)]. This disturbed keratin pattern indicates that the corn epidermis does not have sufficient time to induce typical differentiation markers due to its fast differentiation. These data demonstrate that the mathematical model (Fig. 5) recapitulates human corn morphology as well as epidermal hyperproliferation and rapid differentiation of the corn. Although these experiments do not measure dynamic data as in the mathematical model due to the static images that could be retrieved in the histological sections, the findings are consistent with the model (Fig. 5), where the proliferation and differentiation dynamics are pathological in a subset of keratinocytes, accounting for the corn development.

## Discussion

The numerical results presented above are compatible with our previous results: Using the previous model with a flat, rigid dermis, we have already reported both the stable epidermal structure as in Fig. 1^26^ and the cell supply dependence of the stability of the suprabasal layer^40^. Also, the shape of the dermis, as well as the spatial distribution pattern of stem cells, is qualitatively the same as in the dermal deformation model^29^. These features are preserved in the present integrated model. The reduction of undulation magnitude in the dermis by stiffening is also consistent with the previous model^29^. More importantly, the present integrated model has also revealed that the stiffness could affect the suprabasal layers, as shown in Fig. 4, which can be studied only by treating the epidermis and the deformable dermis simultaneously.

How dermal protrusions arise has been studied in the previous work^29^, where it has been shown that a flat shape of the basement membrane destabilizes due to a buckling instability, and resulting upward protrusions and stem cell distributions are determined by differential adhesion of basal cells. This can be explained as follows: The basement membrane has stretching and bending elasticity. Since cells are adhesive to the basement membrane, cell division on the basement membrane exerts tangential forces on it, creating negative surface tension. Thus a flat shape becomes destabilized due to a buckling instability. To understand why protrusions direct upward and why stem cells are on the top of protrusions, we need to consider differential adhesion. When a cell strongly adhesive to the membrane divide, upward bending (a convex shape) would require a smaller energy cost (because of smaller stretching) than downward bending (a concave shape). The same argument applies to a cell weakly adhesive to the membrane, but since it can also leave the basement membrane, it is energetically more preferable for strongly bound cells to occupy the place with upward protrusions. This scenario has been numerically confirmed^29^.

We have made two major modifications to the previous epidermal model^26^. First, the previous model did not consider the shape change of cells during differentiation. By taking this into account, we have succeeded in producing columnar structures in granular and cornified cells, as shown in Fig. 1, which was not found in the previous model. It is well known that the granular and the cornified layers have columnar structures made of flattened cells^41,42^. We note that the flattening process was also introduced in a different model^5^. Second, in addition to calcium ions, we have assumed that a second factor is released from cells undergoing cornification. In the previous model, we have shown that a localized layer of calcium ions could maintain a well-defined boundary between cornified cells and granular cells. Both the previous and the present models assume that calcium ions released at the time of cornification accelerate cell differentiation, which causes stabilization of the boundary of the cell layer. How this mechanism works has been analytically confirmed by using a reaction-diffusion-advection model^43^. Since the previous model did not distinguish the granular and the spinous cells, however, an additional mechanism is needed to create another boundary between the granular and the spinous layers. Hence we introduced the second factor that can modify differentiation speed differently from calcium ions. The two factors work together to form two boundaries separating the spinous, granular, and cornified layers, as shown in Fig. 1.

In this work, we have modeled proliferative cells by stem cells and TA cells. In order that stem cells do not leave the basal layer, we have assumed that stem cells are bound to the basal layer more strongly than TA cells. Different cell division scenarios, such as a single progenitor compartment model^44^, where a single population of basal cells can divide indefinitely and differentiate with a certain probability, would require a different model for the adhesion of basal cells to the basement membrane. There are studies using agent-based models on the comparison of different stem cell hypotheses^4^ and the mechanism for the maintenance of stem cell niche^45^. It is worth investigating to what degree these modifications to our model would change the stability of the epidermal homeostasis.

Our result on the stiffness of the dermis (Fig. 4) may shed light on photoaging, a physiological senescence process induced by ultraviolet exposure: Ultraviolet destroys elastic fibers in the dermis, leading to dermal stiffness. Photoaging causes changes in the dermal structures, such as flattening of the basement membrane and thinning of the epidermis^46^, accompanied by the decrease in the number and the activity of stem cells^47^. This phenomenon can be accounted for by our model: When the elasticity is lost in the dermis by photoaging, undulations in the basement membrane would be suppressed, with the surface area diminished. Then the cell supply would decrease, resulting in the thinning of the epidermis.

In summary, we have presented an integrated framework for simulating epidermal homeostasis by combining the epidermal structure model^26^ and the dermal deformation model^29^. The previous epidermis model^26^ had already found applications in simulating a thick epidermis equivalent on an undulating substrate^28^ or the epidermal proliferation under the reduced adhesion of progenitor cells to the basement membrane^48^. In this work, by considering the deformation of the dermis, we could also simulate the formation of a corn, which was consistent with the experimental results. Furthermore, our model allows us to infer dynamics that the experiments could not directly observe, which is one of the major advantages of mathematical modeling. Our model is expected to be applied for simulating various diseases entailing structural changes of the dermis.

## Methods

### Histology of human samples

H&E staining and immunohistochemistry for formalin-fixed paraffin-embedded samples were performed on three human corn specimens. The following antibodies were used for immunohistochemistry: anti-K1 (ThermoFisher, Waltham, Massachusetts, USA; 34B4), anti-K6 (ThermoFisher; LHK6B), anti-K10 (Santa Cruz Biotechnology, Dallas, Texas, USA; LHP1), anti-K14 (ThermoFisher; LL002), and anti-Ki-67 (Santa Cruz Biotechnology; MIB-1). Images of immunohistochemistry and H&E sections were captured with a BZ-9000 microscope (Keyence, Tokyo, Japan). For quantification of Ki-67+ cells in the epidermis, the whole areas of the specimens were analyzed using ImageJ (NIH, Bethesda, Maryland, USA). The number of Ki-67+ cells was normalized by the length of the epidermis in each section. The institutional review board of the Hokkaido University Graduate School of Medicine approved all human studies described above (ID: 14-063). The study was conducted according to the Declaration of Helsinki Principles. Participants provided written informed consent.

### Evaluation functions

We define the measures *H*, *G*, and *E* for the granular layer as follows (the same quantities are defined for the cornified layer in the same way). First, we divide the region $0\le x \le L_{x}$ and $0\le y \le L_{y}$ into $M_{1}\times M_{2}$ subregions, with *i*, *j*-subregions defined as $(i-1)\Delta _{x}\le x < i\Delta _{x}$ and $(j-1)\Delta _{y}\le y < j\Delta _{y}$ ($i=1, \dots , M_1$, $j=1, \dots , M_2$) with $\Delta _{x}=L_{x}/M_{1}$ and $\Delta _{y}=L_{y}/M_{2}$. Then we define the thickness $H_{ij}(t)$ in the *i*, *j*-subregion as the total (approximated) volume occupied by the cells divided by the area of the subregion:

1 $$\begin{aligned} H_{ij}(t)=\frac{4\pi R^{3}n_{ij}(t)}{3\Delta _{x}\Delta _{y}}, \end{aligned}$$

where $n_{ij}(t)$ is the number of granular cells. Note that we ignore cell flattening for computing $H_{ij}(t)$. Also, we define the dispersion of cell distribution in the *z* direction is defined as

2 $$\begin{aligned} G_{ij}(t) = z_{ij}^{\mathrm {max}}(t)-z_{ij}^{\mathrm {min}}(t)-H_{ij}(t), \end{aligned}$$

where $z_{ij}^{\mathrm {max}}(t)$ and $z_{ij}^{\mathrm {min}}(t)$ are the maximum and the minimum *z* value of granular cells in the *i*, *j*-subregion, respectively. The mean thickness *H* and the dispersion *G*(*t*) of the whole cell group are given by

3 $$\begin{aligned} G(t)&=\frac{1}{M_{1}M_{2}}\sum ^{M_{1}}_{i=1}\sum ^{M_{2}}_{j=1}G_{ij}(t), \end{aligned}$$

4 $$\begin{aligned} H(t)&=\frac{1}{M_{1}M_{2}}\sum ^{M_{1}}_{i=1}\sum ^{M_{2}}_{j=1}H_{ij}(t). \end{aligned}$$

The spatial modulation of the thickness *E* is defined as the standard deviation of $H_{ij}$:

5 $$\begin{aligned} E(t)= \sqrt{\frac{1}{M_{1}M_{2}}\sum ^{M_{1}}_{i=1}\sum ^{M_{2}}_{j=1}(H(t)-H_{ij}(t))^{2}}. \end{aligned}$$

## Supplementary Information

Supplementary Information.

## Data Availability

The code used in the simulation is available at https://doi.org/10.5281/zenodo.4722355.

## References

[1] Elias, P. M. & Feingold, K. R. Stratum corneum barrier function: definitions and broad concepts. In *Skin Barrier* (eds. Elias, P. M. & Feingold, K. R.) 1-4 (Marcel Dekker, New York, 2005).

[2] Natsuga K (2014). Epidermal barriers. Cold Spring Harb. Perspect. Med..

[3] Schaller G, Meyer-Harmann M (2007). A modelling approach towards epidermal homoeostasis control. J. Theor. Biol..

[4] Li X (2013). Skin stem cell hypotheses and long term clone survival explored using agent-based modelling. Sci. Rep..

[5] Sütterlin T, Tsingos E, Bensaci J, Stamatas GN, Grabe N (2017). A 3D self-organizing multicellular epidermis model of barrier formation and hydration with realistic cell morphology based on EPISIM. Sci. Rep..

[6] Du H (2018). Multiscale modeling of layer formation in epidermis. PLoS Comput. Biol..

[7] Sun T, Adra S, Smallwood R, Holcombe M, MacNeil S (2009). Exploring hypotheses of the action of TGF-$$\beta $$1 in epidermal wound healing using a 3D computational multiscale model of the human epidermis. PLoS ONE.

[8] Adra S, Sun T, MacNeil S, Holcombe M, Smallwood R (2010). Development of a three dimensional multiscale computational model of the human epidermis. PLoS ONE.

[9] Wang Y (2019). A multiscale hybrid mathematical model of epidermal-dermal interactions during skin wound healing. Exp. Dermatol..

[10] Zhang H (2015). Modelling epidermis homoeostasis and psoriasis pathogenesis. J. R. Soc. Interface.

[11] Sakuntabhai A (1999). Mutations in $$\text{ATP}_2$$$$\text{ A}_2$$, encoding a $$\text{ Ca}^{2+}$$ pump, cause Darier disease. Nat. Genet..

[12] Hu Z (2000). Mutations in $$\text{ ATP}_2$$$$\text{ C}_1$$, encoding a calcium pump, cause Hailey–Hailey disease. Nat. Genet..

[13] Meşe G, Richard G, White TW (2007). Gap junctions: Basic structure and function. J. Invest. Dermatol..

[14] Forslind B, Werner-Linde Y, Lindberg M, Pallon J (1999). Elemental analysis mirrors epidermal differentiation. Acta Derm. Venereol..

[15] Mauro T (1998). Acute barrier perturbation abolishes the $$\text{ Ca}^{2+}$$ and $$\text{ K}^{+}$$ gradients in murine epidermis: Quantitative measurement using PIXE. J. Invest. Dermatol..

[16] Denda M, Hosoi J, Ashida Y (2000). Visual imaging of ion distribution in human epidermis. Biochem. Biophys. Res. Commun..

[17] Menon GK, Price LF, Bommannan B, Elias PM, Feingold KR (1994). Selective obliteration of the epidermal calcium gradient leads to enhanced lamellar body secretion. J. Invest. Dermatol..

[18] Elias PM (2002). Modulations in epidermal calcium regulate the expression of differentiation-specific markers. J. Invest. Dermatol..

[19] Cornelissen LH, Oomens CWJ, Huyghe JM, Baaijenset FPT (2007). Mechanisms that play a role in the maintenance of the calcium gradient in the epidermis. Skin Res. Technol..

[20] Adams MP, Mallet DG, Pettet GJ (2012). Active regulation of the epidermal calcium profile. J. Theor. Biol..

[21] Kobayashi A (2014). Mathematical modeling of calcium waves induced by mechanical stimulation in keratinocytes. PLoS ONE.

[22] Grabe N, Neuber K (2005). A multicellular systems biology model predicts epidermal morphology, kinetics and $$\text{ Ca}^2+$$ flow. Bioinformatics.

[23] Proksch E, Brandner JM, Jensen JM (2008). The skin: An indispensable barrier. Exp. Dermatol..

[24] Walker D, Sun T, MacNeil S, Smallwood R (2006). Modeling the effect of exogeneous calcium on keratinocyte and HaCat cell proliferation and differentiation using an agent-based computational paradigm. Tissue Eng..

[25] Sun T (2007). An integrated systems biology approach to understanding the rules of keratinocyte colony formation. J. R. Soc. Interface.

[26] Kobayashi Y, Sawabu Y, Kitahata H, Denda M, Nagayama M (2016). Mathematical model for calcium-assisted epidermal homeostasis. J. Theor. Biol..

[27] Kobayashi, Y. & Nagayama, M. Mathematical model of epidermal structure. In *Applications + Practical Conceptualization + Mathematics = fruitful Innovation: Proceedings of the Forum of Mathematics for Industry 2014* (eds. Anderssen, R. S. et al.) 121-126 (Springer, Tokyo, 2016).

[28] Kumamoto J (2018). Mathematical-model-guided development of full-thickness epidermal equivalent. Sci. Rep..

[29] Kobayashi Y (2018). Interplay between epidermal stem cell dynamics and dermal deformation. npj Comp. Mat..

[30] Hannezo E, Prost J, Joanny JF (2014). Theory of epithelial sheet morphology in three dimensions. Proc. Natl. Acad. Sci. USA.

[31] Basan M, Joanny JF, Prost J, Risler T (2011). Undulation instability of epithelial tissues. Phys. Rev. Lett..

[32] Tallinen T, Biggins JS (2015). Mechanics of invagination and folding: hybridized instabilities when one soft tissue grows on another. Phys. Rev. E.

[33] Nelson MR, King JR, Jensen OE (2013). Buckling of a growing tissue and the emergence of two-dimensional patterns. Math. Biosci..

[34] Varner VD, Gleghorn JP, Miller E, Radisky DC, Nelson CM (2015). Mechanically patterning the embryonic airway epithelium. Proc. Natl. Acad. Sci. USA.

[35] Ben Amar M, Jia F (2013). Anisotropic growth shapes intestinal tissues during embryogenesis. Proc. Natl. Acad. Sci. USA.

[36] Shyer AE (2013). Villification: How the gut gets its villi. Science.

[37] Dunn SJ (2012). A two-dimensional model of the colonic crypt accounting for the role of the basement membrane and pericryptal fibroblast sheath. PLoS Comput. Biol..

[38] Ciarletta P (2013). Buckling instability in growing tumor spheroids. Phys. Rev. Lett..

[39] Swensson O (1998). Specialized keratin expression pattern in human ridged skin as an adaptation to high physical stress. Br. J. Dermatol..

[40] Kobayashi, Y., Sawabu, Y., Ota, S. & Nagayama, M. Mathematical model for epidermal homeostasis. In *Mathematical Progress in Expressive Image Synthesis II* (eds. Ochiai, H. & Anjyo, K.) 119–123 (Springer, Tokyo, 2015).

[41] MacKenzie IC (1969). Ordered structure of the stratum corneum of mammalian skin. Nature.

[42] Christophers E (1971). Cellular architecture of the stratum corneum. J. Invest. Dermatol..

[43] Kobayashi Y, Kitahata H, Nagayama M (2015). Model for calcium-mediated reduction of structural fluctuations in epidermis. Phys. Rev. E.

[44] Clayton E (2007). A single type of progenitor cell maintains normal epidermis. Nature.

[45] Miller, C., Crampin, E. & Osborne, J. Maintaining the stem cell niche in multicellular models of epithelia. preprint at arXiv:1811.10781 (2020). 10.1016/j.jtbi.2021.110807 34119497

[46] Fenske NA, Lober CW (1986). Structural and functional changes of normal aging skin. J. Am. Acad. Dermatol..

[47] Oh J, Lee YD, Wagers AJ (2014). Stem cell aging: Mechanisms, regulators and therapeutic opportunities. Nat. Med..

[48] Watanabe M (2017). Type XVII collagen coordinates proliferation in the interfollicular epidermis. eLife.

