# Supplementary material for: A computational model of the epidermis with the deformable dermis and its application to skin diseases
Source: Sci Rep. 2021 Jun 24;11:13234. doi: 10.1038/s41598-021-92540-1 (PMC8225835; doi:10.1038/s41598-021-92540-1)
Supplement: Supplementary file 1 — Supplementary Information. [file 41598_2021_92540_MOESM1_ESM.pdf]

Supplementary Information

A computational model of the epidermis with the deformable dermis and its application to skin diseases

Kota Ohno, Yasuaki Kobayashi, Masaaki Uesaka, Takeshi Gotoda, Mitsuhiro Denda, Hideyuki Kosumi , Mika Watanabe, Ken Natsuga & Masaharu Nagayama

Details of the mathematical model

Overview

Cell i is assigned internal variables $(S_i(t), \phi_i(t), c_i(t), \kappa_i(t))$, which directly affect the motion of cells: $S_i(t)$ is the state variable, expresses the degree of differentiation; $\phi(t)$ is the phase of the cell division cycle; $c_i(t)$ is the intracellular calcium ions, which affects S_i and ϕ_i; and the expression level of the desmosome $\kappa_i(t)$, which determines the strength of cell-cell adhesion and controls desquamation. These variables are influenced by yet another set of internal variables, as described below.

Both the dermis and the basement membrane are composed of spheres (hereafter referred to as membrane particles or dermal particles) with radius R_{der} and R_{memb}, respectively. The basement membrane particles are connected in the form of a triangular lattice. The motion of dermal or membrane particle i is specified by the position vector $\boldsymbol{r}_i(t)$. An epidermal cell is modeled as a spheroid, whose flattening rate varies depending on S_i. The motion of cell i is specified by the position vector $\boldsymbol{r}_i(t) = (x_i(t), y_i(t), z_i(t))$ and the direction of the short axis $\boldsymbol{n}_i(t)$.

The equations of motion

Let Ω_{der}, Ω_{memb}, Ω_{basal}, Ω_{diff} be the sets of dermal particles, membrane particles, basal layer cells, and differentiated cells, respectively. The positions of these particles are governed by the following equations:

$$\frac{d}{dt}\boldsymbol{r}_i = -\mu\frac{\partial}{\partial \boldsymbol{r}_i}\left(U_{\text{der}} + U_{\text{md}} + U_{\text{wall}}\right), \quad i \in \Omega_{\text{der}}. \tag{1}$$

$$\frac{d}{dt}\boldsymbol{r}_i = -\mu\frac{\partial}{\partial \boldsymbol{r}_i}\left(U_{\text{md}} + U_{\text{mm}} + U_{\text{mc}} + U_{\text{wall}}\right), \quad i \in \Omega_{\text{memb}}, \tag{2}$$

$$\frac{d}{dt}\boldsymbol{r}_i = -\mu\frac{\partial}{\partial \boldsymbol{r}_i}\left(U_{\text{cc}} + U_{\text{mc}}\right), \quad i \in \Omega_{\text{basal}}, \tag{3}$$

$$\frac{d}{dt}\boldsymbol{r}_i = -\mu\frac{\partial}{\partial \boldsymbol{r}_i}U_{cc}, \quad i \in \Omega_{\text{diff}}. \tag{4}$$

Individual interaction functions are explained below.

The dermis

U_{der} represents interactions between dermal particles, which has been modified from the previous work[29]:

$$U_{\text{der}} = \sum_{i,j\in\Omega_{\text{der}}, i<j} K_{\text{d}} u_{\text{d}}\left(\frac{\|\boldsymbol{r}_i - \boldsymbol{r}_j\|}{2R_{\text{der}}}\right), \tag{5}$$

where

$$u_{\text{d}}(x) = \begin{cases} \psi\left(x + \frac{\delta_d}{2R_{\text{der}}}\right) + \frac{K_{\text{sp}}}{2}(x-1)^2 & x < 1 - \frac{\delta_d}{2R_{\text{der}}}, \\ \frac{K_{\text{sp}}}{2}(x-1)^2 + \psi(1) & 1 - \frac{\delta_d}{2R_{\text{der}}} \le x < 1, \\ \psi(x) + \frac{K_{\text{sp}}}{2}(x-1)^2 & 1 \le x < l^*, \\ \psi(l^*) + \frac{K_{\text{sp}}}{2}(l^*-1)^2 & l^* \le x, \end{cases}, \tag{6}$$

$$\psi(x) = \frac{1}{12}x^{-12} - \frac{1}{6}x^{-6}. \tag{7}$$

The function ψ describes weak adhesion when two particles do not overlap ($x > 1$) and the excluded volume effect, i.e., the hard-core repulsion when two cells overlap ($x < 1$). The function $u_{\rm d}(x)$ then describes short-range repulsive and long-range adhesive interactions between two dermal particles; when overlapping ($x < 1$), they first feel an elastic repulsion ($1-\delta_d/2R_{\rm der} \le x < 1$) and then a hard-core repulsion ($x < 1-\delta_d/2R_{\rm der}$). For $x \ge 1$, the two particles feel adhesive interaction with a cutoff range l^*. The parameter $\delta_{\rm d}$ determines the compressibility of the dermis. The stiffness of the dermis can be controlled by varying the strength of the elastic interaction $K_{\rm sp}$. The functional form of $u_{\rm d}(x)$ has been changed from the previous work[29] to incorporate this stiffness control.

The basement membrane

$U_{\rm mm}$ represents the elastic interactions among the membrane particles, which are the same as the previous work[29]:

$$U_{\rm mm} = \sum_{(i,j)\in E_m} \left[K_{\rm str} u_{\rm str}\left(\frac{\|\boldsymbol{r}_i - \boldsymbol{r}_j\|}{2R_{\rm memb}}\right) + K_{\rm bend} u_{\rm bend}\left(\boldsymbol{N}_{ij}^{(1)}, \boldsymbol{N}_{ij}^{(2)}\right)\right] + \sum_{(i,j)\notin E_m} K_{\rm ex} u_{\rm ex}\left(\frac{\|\boldsymbol{r}_i - \boldsymbol{r}_j\|}{2R_{\rm memb}}\right), \tag{8}$$

where E_m represents the set of links of the basement membrane network; $u_{\rm str}$ and $u_{\rm bend}$ describe the stretching and the bending energies assigned to a given link, respectively; The third term describes excluded volume interactions between two membrane particles not connected by a link, which becomes relevant to avoid overlapping in the case of large deformation. The functions $u_{\rm str}$ and $u_{\rm bend}$ are given by

$$u_{\rm str}(x) = \begin{cases} \psi\left(\frac{x}{\delta_m}\right) & x < \delta_m, \\ \frac{\alpha_m}{2\delta_m^2}(x-\delta_m)^2 + \psi(1) & \delta_m \le x < 1, \\ \psi\left(\frac{1+\delta_m - x}{\delta_m}\right) - \frac{\alpha_m(1-\delta_m^2)}{2\delta_m^2} + \frac{\alpha_m(1-\delta_m)}{\delta_m^2}x & 1 \le x, \end{cases} \tag{9}$$

$$u_{\rm bend}\left(\boldsymbol{N}_{ij}^{(1)}, \boldsymbol{N}_{ij}^{(2)}\right) = \frac{1}{2}\left(1 - \boldsymbol{N}_{ij}^{(1)} \cdot \boldsymbol{N}_{ij}^{(2)}\right)^2. \tag{10}$$

Here $u_{\rm str}(x)$ is determined in such a way that the stretching elasticity is linear when the compression of the edge is small ($\delta_m \le x \le 1$ and nonlinear elasticity outside of this range, which prevents indefinite deformation by constant external forces. $\boldsymbol{N}_{ij}^{(1)} = \frac{(\boldsymbol{r}_i-\boldsymbol{r}_j)\times(\boldsymbol{r}_{ij}^{(1)}-\boldsymbol{r}_j)}{\left\|(\boldsymbol{r}_i-\boldsymbol{r}_j)\times(\boldsymbol{r}_{ij}^{(1)}-\boldsymbol{r}_j)\right\|}$ and $\boldsymbol{N}_{ij}^{(2)} = \frac{(\boldsymbol{r}_j-\boldsymbol{r}_i)\times(\boldsymbol{r}_{ij}^{(2)}-\boldsymbol{r}_i)}{\left\|(\boldsymbol{r}_j-\boldsymbol{r}_i)\times(\boldsymbol{r}_{ij}^{(2)}-\boldsymbol{r}_i)\right\|}$ are unit normals for two adjacent triangular regions that are determined by link (i,j); $\boldsymbol{r}_{ij}^{(1)}$ and $\boldsymbol{r}_{ij}^{(2)}$ are the two membrane particles linked to both $\boldsymbol{r}_i$ and $\boldsymbol{r}_j$; both of the normals are defined in such a way that they point outward (i.e., from dermis to epidermis).

$U_{\rm md}$ represents interactions between a membrane particle and a dermal particle, which has been modified from the previous work[29]:

$$U_{\rm md} = \sum_{i\in\Omega_{\rm der}, j\in\Omega_{\rm memb}} \left[\delta_{j,{\rm nmp}(i)} K_{\rm md} u_{\rm md}\left(\frac{\|\boldsymbol{r}_i - \boldsymbol{r}_j\|}{R_i + R_j}\right) + K_{\rm ex} u_{\rm ex}\left(\frac{\|\boldsymbol{r}_i - \boldsymbol{r}_j\|}{R_i + R_j}\right)\right]. \tag{11}$$

The first term represents adhesion of dermal particle i to the nearest membrane particle ${\rm nmp}(i)$; δ is the Kronecker delta. The second term represents excluded volume interactions. The functions are given by

$$u_{\rm md}(x) = \begin{cases} 0 & x < 1, \\ \frac{1}{2}(x-1)^2 & 1 \le x < l^*_{\rm memb}, \\ \frac{1}{2}(l^*_{\rm memb}-1)^2 & l^*_{\rm memb} \le x, \end{cases} \tag{12}$$

$$u_{\rm ex}(x) = \begin{cases} \psi(x) - \psi(1) & x < 1, \\ 0 & 1 \le x, \end{cases} \tag{13}$$

where the adhesive interaction $u_{\rm md}$ has a cutoff at $\|\boldsymbol{r}_i - \boldsymbol{r}_j\| = l^*_{\rm memb}(R_i + R_j)$ and vanishes when the distance exceeds this range. The function $u_{\rm ex}$ expresses only the repulsive interaction.

U_{wall} represents the interaction between a dermal or membrane particle and a boundary wall placed beneath the dermis at $z=0$ through the excluded volume interaction, as in the previous work[29]:

$$U_{\text{wall}} = \sum_{i \in \Omega_{\text{der}}, \Omega_{\text{memb}}} K_{\text{ex}} u_{\text{ex}} \left(\frac{\|\boldsymbol{r}_i - \widehat{\boldsymbol{r}}_i\|}{2R_i} \right), \tag{14}$$

where the position $\widehat{\boldsymbol{r}}_i$ is a mirror image of $\boldsymbol{r}_i$ regarding the boundary wall at $z=0$.

Cell adhesion to the basement membrane

U_{mc} represents interactions between basal cells and the basement membrane[29]:

$$U_{\text{mc}} = \sum_{i \in \Omega_{\text{basal}}, j \in \Omega_{\text{memb}}} \left[K_{\text{ex}} u_{\text{ex}} \left(\frac{\|\boldsymbol{r}_i - \boldsymbol{r}_j\|}{R_i + R_{\text{memb}}} \right) + \delta_{j,\text{nmp(i)}} (1 - \chi_i^{\text{stem}}) K_{\text{ad}} u_{\text{ad}} \left(\frac{\|\boldsymbol{r}_i - \boldsymbol{r}_j\|}{R_i + R_{\text{memb}}} \right) \right.$$
$$\left. + \delta_{j,\text{nmp(i)}} \chi_i^{\text{stem}} K_{\text{bind}} u_{\text{bind}} \left(\frac{\|\boldsymbol{r}_i - \boldsymbol{r}_j\|}{R_i + R_{\text{memb}}} \right) \right]. \tag{15}$$

The first term describes excluded volume interactions between basal cells and membrane particles. The second and the third terms describe adhesion to the basement membrane for transit amplifying (TA) cells and stem cells, respectively, where $\chi_i^{\text{stem}} = 1$ when cell i is a stem cell, and $\chi_i^{\text{stem}} = 0$ for a transit amplifying (TA) cell; and

$$u_{\text{ad}}(x) = \begin{cases} 0 & x < 1, \\ \frac{(x-1)^2}{2} - \frac{(x-1)^4}{4(l^*-1)^2} & 1 \le x < l^*, \\ \frac{(l^*-1)^2}{4} & l^* \le x, \end{cases} \tag{16}$$

$$u_{\text{bind}}(x) = \frac{1}{2}(x-1)^2. \tag{17}$$

The two functions $u_{\text{ad}}(x)$ and $u_{\text{bind}}(x)$ are defined in such a way that only TA cells can leave the basement membrane, whereas stem cells are bound to the basement membrane. For TA cell i, the adhesive force vanishes at $\|\boldsymbol{r}_i - \boldsymbol{r}_{\text{nmp}(i)}\| = l^*(R_i + R_{\text{memb}})$. At this point the TA cell is regarded as differentiated, and S_i starts to increase (explained below).

Cell-cell interaction and cell division

U_{cc} represents the interaction energy between two cells, which consists of two parts:

$$U_{\text{cc}} = \sum_{\substack{i,j \in \Omega_{\text{c}}, i<j \\ (i,j) \notin \Omega_{\text{dp}}}} \left[K_{\text{ex}} u_{\text{ex}} \left(\frac{\|\boldsymbol{r}_i - \boldsymbol{r}_j\|}{R_{ij}^{\text{eff}} + R_{ji}^{\text{eff}}} \right) + K_{\text{ad}}^{(i,j)} u_{\text{ad}} \left(\frac{\|\boldsymbol{r}_i - \boldsymbol{r}_j\|}{R_{ij}^{\text{eff}} + R_{ji}^{\text{eff}}} \right) \right] + \sum_{(i,j) \in \Omega_{\text{dp}}} K_{\text{div}} u_{\text{div}} \left(\|\boldsymbol{r}_i - \boldsymbol{r}_j\| \right), \tag{18}$$

where $\Omega_{\text{c}} = \Omega_{\text{basal}} \cup \Omega_{\text{diff}}$ is the set of cells, and Ω_{dp} is the set of a pair of cells in a cell division process. The first two terms in the bracket describe interactions between cells that are not involved with cell division, which are composed of excluded volume interactions and adhesive interactions. Reflecting its spheroidal shape, a differentiated cell i interacts with another cell j through the effective radii R_{ij}^{eff} and R_{ji}^{eff}, as defined below. The coefficient $K_{\text{ad}}^{(i,j)}$ is modified from the previous work[29], and is given by the mean adhesion strength of two cells i and j:

$$K_{\text{ad}}^{(i,j)} = \frac{1}{2}(\kappa_i(t) + \kappa_j(t)). \tag{19}$$

The differentiation-dependent adhesion strength $\kappa_i(t)$ is described below. Supplementary Figure S1 shows the functional form of the interaction potential between two cells i and j, $K_{\text{ex}} u_{\text{ex}}(x) + \kappa u_{\text{ad}}(x)$ when two cells are of the same kind, $\kappa_i = \kappa_j = \kappa$. Note that, for $x < 1$, $u_{\text{ad}}(x)$ vanishes and the two curves reduce to $u_{\text{ex}}(x)$, the excluded volume interaction.

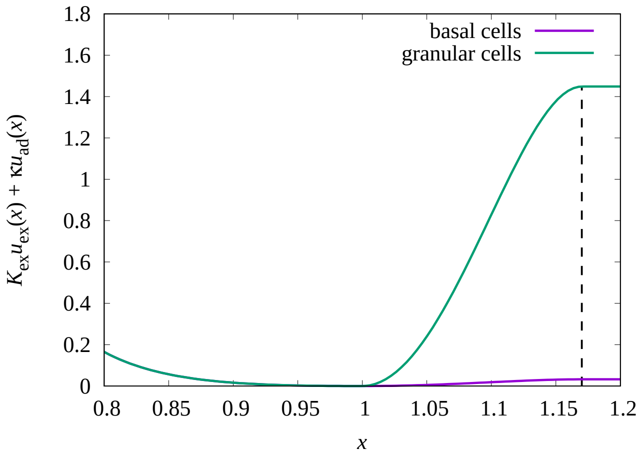

Supplementary Figure 1. Interaction potential between two cells with parameter κ. Plots are shown for two basal cells ($S_i = 0$) and two granular cells ($S_i = S_{SG}$). The adhesion strength κ for $S_i = 0$ and $S_i = S_{SG}$ is obtained from Eq. (29) as $\kappa = 4.5$ and $\kappa = 200.475$, respectively. Vertical dashed line represents the cutoff range of adhesion ($x = l^*$, above which the potential is constant. Note that the two curves completely overlap for $x < 1$, described by $u_{\mathrm{ex}(x)}$.

The third term represents cell division. The cell division process is modeled in the same way as in the previous work[29]: Two dividing cells (i, j) are completely overlapped at the onset of division: they start to be separated by a spring force whose natural length $l(t)$ increases in time from 0. Hence, u_{div} and $l(t)$

$$u_{\mathrm{div}}(x) = \frac{1}{2}(x - l(t))^2, \tag{20}$$

$$\frac{d}{dt}l(t) = \varepsilon_L[2R_{\mathrm{cell}} - l(t)]. \tag{21}$$

The division is complete when $\|\boldsymbol{r}_i(t) - \boldsymbol{r}_j(t)\| > 1.8R_{\mathrm{cell}}$.

Cell division cycle

We assume that stem cells divide infinite number of times and TA cells divide N_{div} times. For both types of cells, a cell division cycle has two stages: in the deterministic stage, the cell division phase $\phi_i(t)$ is governed by[29]

$$\frac{d}{dt}\phi_i = \frac{2\pi}{T_{\mathrm{div}}}\left[1 + \beta_\phi R(c_i - c_0)\right], \tag{22}$$

where $R(x)$ is the ramp function defined by $R(x) = x\Theta(x)$ and Θ is the Heaviside function. Starting from $\phi = 0$, cell division occurs when $\phi(t)$ reaches 2π from 0; Without calcium ions, the period is given by T_{div}. After that, the cell enters a stochastic process, where division occurs following the Poisson process with the division rate γ_{div}. The average waiting time for division in the stochastic process is $\gamma_{\mathrm{div}}^{-1}$. We chose $\gamma_{\mathrm{div}}^{-1} = T_0/3$, so that the overall cell division cycle on average is $(4/3)T_{\mathrm{div}}$. After the cell division, the phase is reset to $\phi = 0$.

Cell differentiation

The degree of differentiation for cell i is expressed by the state variable $S_i(t)$: $S_i(t) = 0$ for a basal cell, $0 < S_i \leq S_{\mathrm{SG}}$ for a spinous cell, $S_{\mathrm{SG}} < S_i \leq S_{\mathrm{SC}}$ for a granular cell, and $S_{\mathrm{SC}} < S_i$ for a cornified cell.

Cell i is regarded as a basal cell when a nonzero adhesive force to the basement membrane exists, as explained

above. When starting differentiation, $S_i(t)$ obeys

$$\frac{d}{dt}S_i = \begin{cases} \alpha_S + \beta_S R(c_i - c_0) + \dfrac{\beta_{\tilde{B}}\tilde{B}_i^n}{B_0 + \tilde{B}_i^n} & 0 < S_i \leq S_{\mathrm{SG}} \\ \alpha_c & S_{\mathrm{SG}} < S_i, \end{cases} \tag{23}$$

where the differentiation speed is assumed to be accelerated by calcium ions $c_i(t)$ and a stimulant $\tilde{B}(t)$. Dynamics of $c_i(t)$ are described below. Dynamics of $\tilde{B}(t)$ are governed by

$$\frac{d}{dt}\tilde{B}_i = -k_{\tilde{B}}\tilde{B}_i + \gamma_{\tilde{B}}\chi(i) + \sum_{j\in\Lambda_i} D_{B_2}\frac{\tilde{B}_j - \tilde{B}_i}{\|\boldsymbol{r}_i - \boldsymbol{r}_j\|^2}. \tag{24}$$

Without $\tilde{B}(t)$, the model of S_i is the same as the previous work[26]. Release of $\tilde{B}(t)$ is assumed to occur ($\chi(i) = 1$) when cell i is a cornified cell that has at least one granular cell adhesively connected to it; otherwise, $\chi(i)$ is set to zero. Λ_i is a set of all cells adhesive to cell i, through which $\tilde{B}(t)$ diffuses.

Cell deformation

When differentiated, cell i starts to flatten into an oblate spheroid with the flattening rate $\alpha_i(t)$, with which the radius of the short and the long axes given by $R_i^{(\min)} = R_i/\alpha_i^2$ and $R_i^{(\max)} = \alpha_i R_i$, respectively, keeping the volume constant. The rate $\alpha_i(t)$ depends on $S_i(t)$:

$$\alpha_i(t) = \frac{1 + \alpha_{\mathrm{flat}}^{(\max)}\beta_{\mathrm{flat}}(S_i/S_{\mathrm{SG}})^{p_{\mathrm{flat}}}}{1 + \beta_{\mathrm{flat}}(S_i/S_{\mathrm{SG}})^{p_{\mathrm{flat}}}}, \tag{25}$$

which increases drastically when cell i becomes a granular cell ($S_i \geq S_{\mathrm{SG}}$).

For differentiated cells, the unit vector of the short axis $\boldsymbol{n}_i(t)$ obeys[28]:

$$\widetilde{\boldsymbol{n}}_i = \frac{\Delta t}{\tau_n}\left(\frac{\boldsymbol{v}_i(t)}{\|\boldsymbol{v}_i(t)\|} - \boldsymbol{n}_i(t)\right) + \boldsymbol{n}_i(t), \tag{26}$$

$$\boldsymbol{n}_i(t + \Delta t) = \frac{\widetilde{\boldsymbol{n}}_i}{\|\widetilde{\boldsymbol{n}}_i\|}, \tag{27}$$

where $\boldsymbol{v}_i(t) = d\boldsymbol{r}_i(t)/dt$. $\boldsymbol{n}_i$ is normalized for each time step Δt.

The effective radius of cell i, when interacting with cell j, depends on $\boldsymbol{n}_i$ and α_i as

$$R_{ij}^{(\mathrm{eff})} = \sqrt{(R_i^{(\min)}\cos\theta_{ij})^2 + (R_i^{(\max)}\sin\theta_{ij})^2}, \tag{28}$$

where $\cos\theta_{ij} = ((\boldsymbol{r}_i - \boldsymbol{r}_j)/||\boldsymbol{r}_i - \boldsymbol{r}_j||)\cdot\boldsymbol{n}_i$. Note thet $R_{ij}^{\mathrm{eff}} \neq R_{ji}^{\mathrm{eff}}$ in general. For basal cell i, $R_i^{(\min)} = R_i^{(\max)} = R_i$ and the effective radius is $R_{ij}^{(\mathrm{eff})} = R_i$.

Desquamation

In the basal, spinous, and granular layers, the adhesion strength κ_i depends on differentiation state S_i:

$$\kappa_i(t) = \frac{\kappa^{(0)} + \kappa^{(\max)}\beta_{\mathrm{adh}}\left(\dfrac{S_i}{S_{\mathrm{SG}}}\right)^{p_{\mathrm{adh}}}}{1 + \beta_{\mathrm{adh}}\left(\dfrac{S_i}{S_{\mathrm{SG}}}\right)^{p_{\mathrm{adh}}}}. \tag{29}$$

In the cornified layer, we adopt the following desquamation model[49]. Cornified cells adhere to each other by corneodesmosomes. Corneodesmosomes are decomposed by degradation enzyme kallikreins (KLKs), which

are expressed and released when cells differentiate from a granular cell to a cornified cell. The activity of KLKs is inhibited by binding with Kazal-type-related inhibitor (LEKTI), depending on the pH gradient in the cornified layer: binding is strong when pH is high as in the deep cornified layer and weak when pH is low as near the surface of the cornified layer. As increasing KLKs, corneodesmosomes decompose and cornified cells peel off from the epidermis.

Hence, in the cornified layer, we consider the following model for the adhesion strength κ_i depending on the released KLKs, denoted by $K_i^{(\mathrm{r})}$:

$$\frac{d}{dt}\kappa_i = -\frac{k_{\mathrm{desq}}^{(\mathrm{max})}(K_i^{(\mathrm{r})})^{p_{\mathrm{desq}}}}{\beta_{\mathrm{desq}} + (K_i^{(\mathrm{r})})^{p_{\mathrm{desq}}}}\kappa_i. \tag{30}$$

Desquamation occurs when $\kappa_i \le K_{\mathrm{off}}$ and fewer than N_{conn} cells are adhesive to cell i.

The dynamics of $K_i^{(\mathrm{r})}$ are described as follows. In the granular layer, we consider the produced and released KLKs, $K_i^{(\mathrm{p})}$ and $K_i^{(\mathrm{r})}$, respectively; and produced and released LEKTI, $L_i^{(\mathrm{p})}$ and $L_i^{(\mathrm{r})}$, respectively. Their dynamics are dependent on S_i and c_i:

$$\frac{d}{dt}K_i^{(\mathrm{p})} = (K_{\mathrm{max}}^{(\mathrm{p})} - K_i^{(\mathrm{p})}) f_{\mathrm{prod}}(c_i, S_i) - K_i^{(\mathrm{p})} f_{\mathrm{rel}}(c_i, S_i), \tag{31}$$

$$\frac{d}{dt}K_i^{(\mathrm{r})} = K_i^{(\mathrm{p})} f_{\mathrm{rel}}(c_i, S_i), \tag{32}$$

$$\frac{d}{dt}L_i^{(\mathrm{p})} = (L_{\mathrm{max}}^{(\mathrm{p})} - L_i^{(\mathrm{p})}) f_{\mathrm{prod}}(c_i, S_i) - L_i^{(\mathrm{p})} f_{\mathrm{rel}}(c_i, S_i), \tag{33}$$

$$\frac{d}{dt}L_i^{(\mathrm{r})} = L_i^{(\mathrm{p})} f_{\mathrm{rel}}(c_i, S_i), \tag{34}$$

where the constant $K_{\mathrm{max}}^{(\mathrm{p})}$ and $L_{\mathrm{max}}^{(\mathrm{p})}$ mean the maximum concentration of intercellular KLKs and LEKTI, respectively, and f_{prod} and f_{rel} describe the production and release of KLKs and LEKTI, respectively:

$$f_{\mathrm{prod}}(c, S) = \frac{k_{\mathrm{prod}}}{4}\left(1 + \tanh\left(\frac{S - S_{\mathrm{SG}}}{\sigma_s}\right)\right)\left(1 + \tanh\left(\frac{c^* - c}{\sigma_c}\right)\right), \tag{35}$$

$$f_{\mathrm{rel}}(c, S) = \frac{k_{\mathrm{rel}}}{4}\left(1 + \tanh\left(\frac{S - S_{\mathrm{SG}}}{\sigma_s}\right)\right)\left(1 + \tanh\left(\frac{c - c^*}{\sigma_c}\right)\right). \tag{36}$$

Here the production and the release start to increase when a cell becomes a granular cell ($S > S_{SG}$). Normally, in the granular layer, calcium levels are small ($c < c^*$), so that the production is dominant; when c_i exceeds c^* due to calcium excitation occurs during cornification, the production stops and the release becomes dominant.

In the cornified layer, $K_i^{(\mathrm{r})}$, $L_i^{(\mathrm{r})}$, and $K_i^{(\mathrm{bind})}$, the concentration of the KLKs bound to LEKTI, are considered. The process of binding and decomposition of KLKs and LEKTI are described by

$$\frac{d}{dt}K_i^{(\mathrm{bind})} = -f_{\mathrm{pH}}(\tilde{S}_i)K_i^{(\mathrm{bind})} + \widehat{f}_{\mathrm{pH}}(\tilde{S}_i)K_i^{(\mathrm{r})}L_i^{(\mathrm{r})}, \tag{37}$$

$$\frac{d}{dt}K_i^{(\mathrm{r})} = f_{\mathrm{pH}}(\tilde{S}_i)K_i^{(\mathrm{bind})} - \widehat{f}_{\mathrm{pH}}(\tilde{S}_i)K_i^{(\mathrm{r})}L_i^{(\mathrm{r})} + \sum_{j \in \Lambda_i^{\mathrm{corn}}} D_{\mathrm{KLKs}}\frac{K_j^{(\mathrm{r})} - K_i^{(\mathrm{r})}}{\|\boldsymbol{r}_i - \boldsymbol{r}_j\|^2}, \tag{38}$$

$$\frac{d}{dt}L_i^{(\mathrm{r})} = f_{\mathrm{pH}}(\tilde{S}_i)K_i^{(\mathrm{bind})} - \widehat{f}_{\mathrm{pH}}(\tilde{S}_i)K_i^{(\mathrm{r})}L_i^{(\mathrm{r})} + \sum_{j \in \Lambda_i^{\mathrm{corn}}} D_{\mathrm{LEKTI}}\frac{L_j^{(\mathrm{r})} - L_i^{(\mathrm{r})}}{\|\boldsymbol{r}_i - \boldsymbol{r}_j\|^2}, \tag{39}$$

where $\tilde{S}_i = (S_i - S_{\mathrm{SC}})/S_{\mathrm{d}}$, $\Lambda_i^{\mathrm{corn}}$ is the set of cornified cells adhesive to cell i, and

$$f_{\mathrm{pH}}(x) = k^{(\max)} \frac{x^p}{\beta + x^p}, \tag{40}$$

$$\widehat{f}_{\mathrm{pH}}(x) = \widehat{k}^{(\max)} \left(1 - \frac{x^{\widehat{p}}}{\widehat{\beta} + x^{\widehat{p}}}\right). \tag{41}$$

We have assumed that the diffusion of the KLKs-LEKTI complex is negligible and that, when a cell moves outward (as S_i becomes larger) $K_i^{(\mathrm{bind})}$ decreases and both $K_i^{(\mathrm{r})}$ and $L_i^{(\mathrm{r})}$ increase, due to the decrease of pH, which are described by $f_{\mathrm{pH}}(x)$ and $\widehat{f}_{\mathrm{pH}}(x)$. Therefore, in the deep cornified layer, the LEKTI-bound KLKs are dominant, whereas near the surface of the cornified layer, isolated KLKs become dominant, which decreases the adhesion strength κ_i and induced desquamation.

Calcium dynamics

The model for calcium dynamics is the same as the previous work[26]. The dynamics of $c_i(t)$ (intercellular calcium), $A(t, \boldsymbol{x})$ (the extracellular ATP concentration), $P_i(t)$ (the concentration of IP_3), $h_i(t)$ (the inactivation factor), $w_{ij}(t)$ (the activity of the gap-junctions between cell i and cell j), and $B_i(t)$ (the stimulant substance) is governed by

$$\frac{\partial A}{\partial t} = d_A \Delta A - K_{aa} A + \Omega_{\mathrm{c}} G\left(\boldsymbol{r}, \boldsymbol{r}_i, \frac{dc_i}{dt}\right), \tag{42}$$

$$\frac{dP_i}{dt} = \sum_{j \in \Lambda_i} d_P w_{ij} I_n(S_i)(P_j - P_i) + F_P(A, S_i) - K_{pp} P_i, \tag{43}$$

$$\frac{dc_i}{dt} = \sum_{j \in \Lambda_i} d_c w_{ij} I_n(S_i)(c_j - c_i) + F_c(P_i, c_i, h_i, B), \tag{44}$$

$$\frac{dh_i}{dt} = \frac{1}{\tau_h(S_{k_i})} F_h(c_i, h_i), \tag{45}$$

$$\frac{dw_{ij}}{dt} = \frac{1}{\tau_w} F_w(w_{ij}, c_i, c_j), \tag{46}$$

$$\frac{dB_i}{dt} = -k_B B_i + \gamma_B \chi(i) + \sum_{j \in \Lambda_i} D_B \frac{B_j - B_i}{\|\boldsymbol{r}_i - \boldsymbol{r}_j\|^2}, \tag{47}$$

where

$$G\left(\boldsymbol{r},\boldsymbol{r}_i,\frac{dc_i}{dt}\right)=K_{ac}\Theta(R_i-\|\boldsymbol{r}-\boldsymbol{r}_i\|)R\left(\frac{dc_i}{dt}\right), \tag{48}$$

$$I_n(S_i)=\frac{1}{2}\left(1+\tanh\left(\frac{S_i-S_{SG}}{\delta_I}\right)\right), \tag{49}$$

$$F_P(A,S_i)=K_{pa}(S_i)\frac{A}{H_0+A}, \tag{50}$$

$$K_{pa}(S_i)=k_g+\frac{k_s-k_g}{2}\left(1+\tanh\left(\frac{S_{SG}-S_i}{\delta_k}\right)\right), \tag{51}$$

$$F_c(P,c,h,B)=K_f\left(\mu_0+\frac{\mu_1 P}{K_\mu+P}\right)\left(\alpha_0+\frac{(1-\alpha_0)c}{K_1+c}\right)h-\frac{\gamma c}{K_\gamma+c}+\beta_c+\frac{K_{bc}B^2}{H_b+B^2}, \tag{52}$$

$$\tau_h(S_i)=\tau_g+\frac{\tau_s-\tau_g}{2}\left(1+\tanh\left(\frac{S_{SG}-S_i}{\delta_\tau}\right)\right), \tag{53}$$

$$F_h(c,h)=\frac{K_2^2}{K_2^2+c^2}-h, \tag{54}$$

$$F_w(w_{ij},c_i,c_j)=-w_{ij}+\frac{1}{2}\left(1+\tanh\left(\frac{w_{d0}-|c_i-c_j|}{\varepsilon_{w0}}\right)\right). \tag{55}$$

Here ATP is released by calcium excitation described by the function G, and diffuses in the extra-cellular regions. IP_3 increase is caused by ATP as described in F_P and diffuses through gap junctions, which appear when cells differentiate into granular cells. Calcium excitation is caused by the presence of IP_3 and a stimulant B and suppressed by the inactivation factor h_i as in $F_c(P,c,h,B)$. The conductivity of the gap junctions w_{ij} is determined by calcium concentrations. The stimulant B is released upon cornification ($\chi(i)=1$) and diffuses into adjacent cells. When calculating $A(t,x)$, we impose periodic boundary conditions in the x and y directions and the Neumann boundary condition in the z direction.

Intercellular lipids

The model for intercellular lipids are the same as the previous work[26]. The production and release of lipids by cell i, $v_i^{(\mathrm{p})}$ and $v_i^{(\mathrm{r})}$, respectively, depend on S_i and c_i:

$$\frac{d}{dt}v_i^{(\mathrm{p})}=(v_{\max}-v_i^{(\mathrm{p})})f^{(\mathrm{p})}(c_i,S_i)-v_i^{(\mathrm{p})}f^{(\mathrm{r})}(c_i,S_i), \tag{56}$$

$$\frac{d}{dt}v_i^{(\mathrm{r})}=v_i^{(\mathrm{p})}f^{(\mathrm{r})}(c_i,S_i), \tag{57}$$

where

$$f^{(\mathrm{p})}(c_i,S_i)=\frac{k^{(\mathrm{p})}}{4}\left(1+\tanh\left(\frac{S_i-S_{\mathrm{SG}}}{\sigma_{r1}}\right)\right)\left(1+\tanh\left(\frac{c^*-c_i}{\sigma_{r2}}\right)\right), \tag{58}$$

$$f^{(\mathrm{r})}(c_i,S_i)=\frac{k^{(\mathrm{r})}}{4}\left(1+\tanh\left(\frac{S_i-S_{\mathrm{SG}}}{\sigma_{r1}}\right)\right)\left(1+\tanh\left(\frac{c_i-c^*}{\sigma_{r2}}\right)\right). \tag{59}$$

Thus, the production of lipids starts when cell i becomes a granular cell ($S_i\geq S_{\mathrm{SG}}$); when the calcium level $c_i(t)$ exceeds c^*, the production ceases and the produced lipids start to be released.

Simulation setup

The system size we consider is $[0,L_x]\times[0,L_y]\times[0,L_z]$, with periodic boundaries in x and y directions. In z direction, a boundary wall is placed at $z=0$, with which dermal particles interact through U_{wall} described above. The L_z value is chosen so that the whole epidermis never reaches $z=L_z$. N_{der} particles are randomly packed on the $z=0$ plane,

and the basement membrane composed of N_{memb} particles forming a triangular lattice is placed on top of it. N_{stem} stem cells are placed on the initially flat basement membrane. In numerical simulations, we have used two sets of system size: $L_x = 150$ [µm], $L_y = 150$ [µm], $L_z = 300$ [µm], $N_{\mathrm{der}} = 7200$, $N_{\mathrm{memb}} = 16560$, and $N_{\mathrm{stem}} = 16$ for Figures 1, 2, 3, and 4; and $L_x = 300$ [µm], $L_y = 300$ [µm], $L_z = 625$ [µm], $N_{\mathrm{der}} = 115200$, $N_{\mathrm{memb}} = 66240$, and $N_{\mathrm{stem}} = 64$ for Figure 5. The simulation time step is $\Delta t = 0.01$. We adopt multi-scale simulations, which separates timescales for calcium dynamics [Eqs. (42)-(46)] and all others (main dynamics)[26]. We compute the main dynamics with a constant calcium level. When 20 cells undergo cornification, we switch to the computation of calcium dynamics until reaching a steady state. Then we return to the main dynamics with a renewed calcium level.

Parameter values used in the model equations are listed in Supplementary Table.1. Parameters that appear in the equations of motion for particles and cells are taken from Reference 29, and those in cell division cycle, cell differentiation, and calcium dynamics are taken from Reference 26. In these references, parameter values have been chosen so that numerical simulations could produce dermal protrusions with reasonable sizes and epidermal layers with well-defined boundaries. In this work, we have chosen smaller sizes of dermal particles and membrane particles, which resulted in the change of interaction strengths regarding the dermis and the basement membrane. To obtain numerical results consistent with the previous work, we modified parameter values in Reference 29. For the parameters that do not appear in these references, their values have been determined so that we could obtain reasonable numerical results. We should note that the parameter values in this work have not been directly verified by experiments.

We have chosen the non-dimensional unit of force by setting the mobility $\mu = 1$, whereby the unit of other parameters is expressed by length [µm] and time [h].

Supplementary Table 1. Model parameters.

parameter name	value	comment
R_{der}	2.5 [µm]	modified from Ref. 29
R_{memb}	2.5 [µm]	modified from Ref. 29d
R_{max}	5.0 [µm]	modified from Ref. 29
K_{d}	1.125 [$\mu m^2 h^{-1}$]	modified from Ref. 29
K_{sp}	0.08 (normal) or 20.48 (stiffened)	assumed
δ_d	$0.4 \times R_{\mathrm{der}}$	modified from Ref. 29
l^*	1.17	modified from Ref. 29
K_{str}	1.125 [$\mu m^2 h^{-1}$]	modified from Ref. 29
K_{bend}	281.25 [$\mu m^2 h^{-1}$]	modified from Ref. 29
K_{ex}	1.125 [$\mu m^2 h^{-1}$]	modified from Ref. 29
δ_{m}	0.25	modified from Ref. 29
a_{m}	0.16	modified from Ref. 29
K_{md}	6750 [$\mu m^2 h^{-1}$]	modified from Ref. 29
l^*_{memb}	1.1	modified from Ref. 29
K_{ad}	590.625 [$\mu m^2 h^{-1}$]	modified from Ref. 29
K_{bind}	2531.25 [$\mu m^2 h^{-1}$]	modified from Ref. 29
K_{div}	22.5 [h^{-1}]	modified from Ref. 29
ε_{L}	0.63 [h^{-1}]	modified from Ref. 29
T_0	4.0 [day]	modified from Ref. 29
β_ϕ	50.0	modified from Ref. 26
c_0	0.122	Ref. 26
S_{SG}	3.0	modified from Ref. 26
S_{SC}	22.0	Ref. 26
α_S	2.025×10^{-5} [h^{-1}]	modified from Ref. 26

β_S	0.536625 [h^{-1}]	modified from Ref. 26
$\beta_{\tilde{B}}$	0.010125 [h^{-1}]	assumed
n	10.0	assumed
B_0	1.0	assumed
α_c	0.0225 [h^{-1}]	modified from Ref. 26
$k_{\tilde{B}}$	0.135 [h^{-1}]	assumed
$\gamma_{\tilde{B}}$	9.0 [h^{-1}]	assumed
$D_{\tilde{B}}$	11.25 [$\mu m^2 h^{-1}$]	assumed
$\alpha_{\text{flat}}^{(\max)}$	1.4	assumed
β_{flat}	1.0	assumed
p_{flat}	7.0	assumed
τ_n	222.222 [h]	assumed
$\kappa^{(0)}$	4.5 [$\mu m^2 h^{-1}$]	assumed
$\kappa^{(\max)}$	984.375 [$\mu m^2 h^{-1}$]	assumed
β_{adh}	0.25	assumed
p_{adh}	10.0	assumed
$k_{\text{desq}}^{(\max)}$	0.18 [h^{-1}]	assumed
p_{desq}	6.0	assumed
β_{desq}	5.0	assumed
K_{off}	337.5 [$\mu m^2 h^{-1}$]	assumed
N_{conn}	16	assumed
$K_{\max}^{(p)}$	1.5	assumed
$L_{\max}^{(p)}$	1.5	assumed
k_{prod}	0.225 [h^{-1}]	assumed
k_{rel}	0.45 [h^{-1}]	assumed
σ_S	0.1	assumed
σ_c	0.001	assumed
c^*	0.35	assumed
D_{KLKs}	0.1125 [$\mu m^2 h^{-1}$]	assumed
D_{LEKTI}	0.1125 [$\mu m^2 h^{-1}$]	assumed
$k^{(\max)}$	0.1575 [h^{-1}]	assumed
p	8.0	assumed
β	2.5	assumed
$\widehat{k}^{(\max)}$	1.8 [h^{-1}]	assumed
$\widehat{p}$	4.0	assumed
$\widehat{\beta}$	0.005	assumed
S_d	7.56	assumed
d_A	1.0	Ref. 26
K_{aa}	0.5	Ref. 26
K_{ac}	0.002	Ref. 26
d_p	0.1	Ref. 26
K_{pp}	0.3	Ref. 26
H_0	0.5	Ref. 26
k_g	4.0	Ref. 26
k_s	6.0	Ref. 26

δ_k	1.0	Ref. 26
d_c	0.01	Ref. 26
δ_I	1.5	Ref. 26
K_f	8.1	Ref. 26
μ_0	0.567	Ref. 26
μ_1	0.1	modified from Ref. 26
K_μ	0.05	Ref. 26
α_0	0.11	Ref. 26
K_1	0.7	Ref. 26
γ	2.0	Ref. 26
K_γ	0.1	Ref. 26
β_c	0.02	Ref. 26
K_{bc}	0.48	modified from Ref. 26
H_b	0.01	Ref. 26
τ_g	0.2	Ref. 26
τ_s	1.0	Ref. 26
δ_τ	1.0	Ref. 26
K_2	0.7	Ref. 26
τ_w	1.0	Ref. 26
w_{d0}	0.1	Ref. 26
ε_{w0}	0.1	Ref. 26
k_B	0.18 [h^{-1}]	modified from Ref. 26
γ_B	9.0 [h^{-1}]	modified from Ref. 26
D_B	2.8125×10^{-3} [$\mu\mathrm{m}^2\mathrm{h}^{-1}$]	modified from Ref. 26
$v_{\max}^{(p)}$	1.0	Ref. 26
$k^{(\mathrm{p})}$	0.25 [h^{-1}]	assumed
$k^{(\mathrm{r})}$	0.45 [h^{-1}]	assumed
σ_{r1}	0.1	assumed
σ_{r2}	0.001	assumed

References

26. Kobayashi, Y., Sawabu, Y., Kitahata, H., Denda, M. & Nagayama, M. Mathematical model for calcium-assisted epidermal homeostasis. *J. Theor. Biol.* **397**, 52-60 (2016).

28. Kumamoto, J. et al. Mathematical-model-guided development of full-thickness epidermal equivalent. *Sci. Rep.* **8**, 17999 (2018).

29. Kobayashi. Y. et al, Interplay between epidermal stem cell dynamics and dermal deformation. *npj Comp. Mat.* **4**, 45 (2018).

49. Ishida-Yamamoto, A. & Kishibe, M. Involvement of corneodesmosome degradation and lamellar granule transportation in the desquamation process. *Med. Mol. Morphol.* **44**, 1-6 (2011).
